# List of Diseases at the Bath City Dispensary, from August 1, to September 1, 1801

**Published:** 1801-10

**Authors:** W. White


*List of Diseases at the Bath City Dispensary, from August* 1, *to September* 1, 1801.

| | | | |
|---|---|---|---|
| Anaſarca - - - - - - - | 3 | Rheumatiſm Acute - - - | 15 |
| Cynanche Maligna - - | 2 | Scalded Leg - - - - - - | 1 |
| Contuſion - - - - - - | 1 | Scarlatina Anginoſa - - | 1 |
| Cholera - - - - - - - | 2 | Synochus Bilioſa - - - | 55 |
| Conſtipatio - - - - - - | 1 | ——— Pleuritica - - | 5 |
| Eruptiones - - - - - - | 3 | ——— Enteritica - - | 2 |
| Febris Simplex - - - - | 5 | ——— Nephritica - - | 1 |
| —— Quotidiana - - - | 1 | ——— Cholerica - - | 7 |
| Gaſtrodynia - - - - - - | 1 | ——— cum Hæmorrhagia | 1 |
| Hæmoptyſis - - - - - - | 1 | ——— cum Paraplegia - | 1 |
| Hectica - - - - - - - - | 1 | ——— Dyſenterica - - | 2 |
| Hernia - - - - - - - - | 1 | ——— Icterica - - - | 4 |
| Herpes - - - - - - - - | 2 | Syphilis - - - - - - - - | 1 |
| Leucorrhœa - - - - - - | 1 | Scrofula - - - - - - - | 3 |
| Menorrhagia - - - - - | 2 | Shingles - - - - - - - | 1 |
| Ophthalmia - - - - - - | 1 | Tumor - - - - - - - - | 1 |
| Phthiſis - - - - - - - | 4 | Tic Doloreux - - - - - | 1 |
| Pertuſſis - - - - - - - | 1 | Ulcerated Legs - - - - | 5 |
| Pleuritis - - - - - - - | 1 | Vermes - - - - - - - - | 1 |
| Prolapſus Ani - - - - - | 1 | Variola - - - - - - - | 5 |
| Pſora - - - - - - - - | 4 | | |

W. WHITE.

CRITICAL

